# Web-Based Information on the Treatment of Dental Hypomineralization

**DOI:** 10.7759/cureus.45840

**Published:** 2023-09-24

**Authors:** Hattan Zaki, Ismail Abdouh, Amnah Algarni, Rahaf Almukhlifi, Somayah Sanad, Muath Alassaf, Mahir Mirah

**Affiliations:** 1 Oral Basic and Clinical Sciences Department, College of Dentistry, Taibah University, Madinah, SAU; 2 Restorative Dental Sciences Department, College of Dentistry, Taibah University, Madinah, SAU; 3 Oral and Maxillofacial Pathology Department, The State University of New York, Buffalo, USA; 4 Dentistry Department, Prince Mohammad Bin Abdulaziz Hospital, Madinah, SAU; 5 Orthodontics Department, College of Dentistry, Taibah University, Madinah, SAU

**Keywords:** jama, smog, fres, fkgl, discern tool, patient education, online health information, dental hypomineralization

## Abstract

Purpose: To categorise and evaluate the quality and readability of the web-based information about the treatment of the variety of forms of dental hypomineralization.

Methods: An internet search using two different search terms regarding treating dental hypomineralization was conducted using the Google search engine. The first 100 websites from each search were analysed. Data recorded included DISCERN instrument scores, the Journal of the American Medical Association (JAMA) benchmarks, and the Health on the Net seal (HON). Flesch Reading Ease Scores (FRES), Flesch-Kincaid Grade Level (FKGL), the Simplified Measure of Gobbledygook Index (SMOG), and the Coleman-Liau index were calculated to assess readability.

Results: A search for "Treatment of hypomineralized teeth" on Google yielded 48,500 results. After excluding irrelevant websites, only 25 were evaluated based on affiliation with universities/medical centers, non-profit organizations, commercial entities, or government agencies. The majority of the content was medical facts presented as text and visuals such as images and videos. The study found that the scores for questions about the benefits and risks of treatment were low, while alternative treatments had high scores. Only one website met the HON code criteria, and a minority of websites achieved JAMA benchmarks. The readability ratings varied across different tests used in the study.

Conclusion: Most websites had university or medical center affiliation but only partially related to the specialty. Two-thirds of websites used images. The online information was inaccurate, poor quality, and hard to read for the average person. Dental professionals should be aware of this information's quality and work to improve it.

## Introduction

Enamel, dentin, and cementum are the three hard tissues forming the tooth, formed by specialized cells. The formation of the tooth is through biological and cellular complex pathways that are controlled by genes and affected by environmental and epigenetic factors. Alterations in genes controlling tooth development result in reduced quantity and quality of the hard tissues forming the tooth. According to the affected gene, the defect will result in dental malformation or other organs will also be involved [1].

Amelogenesis imperfecta (AI), dentinogenesis imperfecta (DI), dental fluorosis (DF), and molar incisor hypomineralization (MIH), are all examples of structural malformation affecting the quality and the quantity of the hard tissues forming the tooth [2]. The prevalence of AI, DI, DF and MIH are estimated to be 0.14%, 4-70%, 2.8% and 40%, respectively [3]. Recent studies have reported that the adverse side effects of teeth with a compromised structure may include poor aesthetics, tooth sensitivity, pulp exposure, and increased risk of caries and tooth wear [1]. Affected individuals often have one of the aforementioned side effects that may negatively impact a patient’s quality of life [4]. Moreover, restorative treatment of the affected teeth is considered challenging and can negatively impact aesthetics and adversely affect a patient’s emotional and social life [4,5]. The failure rate of the restorative treatment on the affected teeth is as high as 43%, as reported by the literature [2].

Reducing the risk mentioned before in both enamel defects (AI, DF, and MIH) and dentin defects (e.g., DI) requires early diagnosis, preventive care, and regular follow-ups. To reduce the caries risk, topical fluoride application is recommended every three to six months. Since the tooth structure is weak and prone to fracture under slight masticatory force, full coverage restoration is advised as soon as the tooth erupts. In addition, children with a family history of amelogenesis imperfecta or with a systemic disease known to be related to dental malformation should be screened early as soon as the teeth erupt [1].

In regard to the chronic nature of the disease and previously mentioned side effects, these malformations are common among related populations hence individuals with enamel or dentin malformation are likely to require or wish to have the appropriate knowledge to help them to correctly seek the required advice along with increasing their awareness about the treatment options and perhaps the complications of the disease and the suggested therapeutic interventions.

In this regard, patients usually consider the Worldwide Web as one of the most approachable sources of healthcare information and patient self-education. Although such online information is easily accessible and plentiful, there are concerns regarding the availability of inaccuracy, poor quality, and difficult readability of the related information sources that may augment the risk of consumption of low-quality related information, hampering the person-professional relationship. Thus, online information could be misleading and hence hinder patient’s participation in the clinical decisions regarding their healthcare needs and the risks and benefits of the treatment options being considered.

Therefore, with the increasing patients’ esthetic demands and the possible complications that can arise from these dental malformations and the need for early diagnosis and treatment intervention, it is crucial to assess the quality and readability of web-based information concerning dental hypomineralization, hence the aim of the present study was to categorise and evaluate the quality and readability of the web-based information about the treatment of the variety of forms of dental hypomineralization including AI, DI, DF and MIH.

## Materials and methods

Search

An online search started in June 2023 using Google.com [6] using two different search terms (treatment of enamel hypomineralization and treatment of hypomineralized teeth). Although it is common for less than 25% of individuals to explore search results beyond the first page on Google.com, the initial 100 websites were included in the study to ensure a comprehensive evaluation of the available online information [7]. The duplications and screened for any non-operative link were removed. We excluded scientific articles, book reviews, irrelevant content, broken links, non-English language links, promotional product websites, membership-based websites, discussion groups, video feeds and online medical dictionaries. The remaining websites were sorted based on Ni Riordain and McCreary's [8] definition which included affiliation (profit organization, university/medical center, and government), specialization (related to the treatment of dental hypomineralization either exclusively or partly), content type (medical facts, clinical trials, question and answer sections and human interest stories), and content presentation (including images, videos, and audio).

Quality assessment

Before data extraction, two reviewers underwent training, and a third reviewer resolved disagreements. The quality was assessed independently using two instruments, the DISCERN instrument [9] and the Journal of the American Medical Association (JAMA) benchmarks [10]. The Health on the Net (HON) seal was recorded.

The DISCERN instrument, comprising 16 items developed by the University of Oxford, was utilized to assess the reliability of online content and its treatment information. This tool evaluates both the overall reliability of information and specific treatment details. The first eight questions explore reliability and the next seven questions specific details of treatment with an additional question for an overall rating of the material’s quality. The questions are rated on a numerical scale from 1 to 5 (1 = very poor, 2 = poor, 3 = moderate, 4 = good, 5 = excellent) [9].

The JAMA benchmarks were used to determine the readability and accuracy of websites, which include the clarity of authorship of medical content, attributions, statements of disclosure, and indication of currency [10].

Health on the Net (HON) is a non-profit organization that was founded in 1995 with the aim of helping people assess the credibility of medical information and sources available online. Websites that meet criteria stating the qualifications of authors and differentiating between advertising and editorial content are eligible to display the HON seal.

Readability assessment

To understand the written texts, a readability assessment is applied which is used to determine the reading comprehension level of a person by a systematic formula [11]. There were four measurements used to assess the readability of the texts: the Flesch Reading Ease Scores (FRES), Flesch-Kincaid Grade Level (FKGL), the Simplified Measure of Gobbledygook Index (SMOG), and the Coleman-Liau index (CLI).

The FRES is based upon a formula that incorporates the average sentence length and the average number of syllables per word and the outcome score is a number ranging from 0 to 100. The higher the score, the easier the passage is to read [12]. For example, a score above 90 reflects that the text is very easy to read by an average fifth-grade student while scores between 60 and 70 are considered to be plain English and easily to understood by eighth- and ninth-grade students. Finally, scores between 50 and 30 are difficult to read and need a college student to understand.

The FKGL is an improvement of FRES and is the average number of words per syllables and sentence. The SMOG index is the number of polysyllabic words per sentence which is considered to determine the level of education needed to understand a written piece. However, the CLI depends on characters per word and sentence length.

Two independent reviewers, SA and RA, assessed text readability to ensure reliability and consistency in our evaluation. A website (www.readabilityformulas.com) [13] was used to determine the difficulty level in understanding a text by utilizing an automated formula.

## Results

Available websites

**Figure 1:**
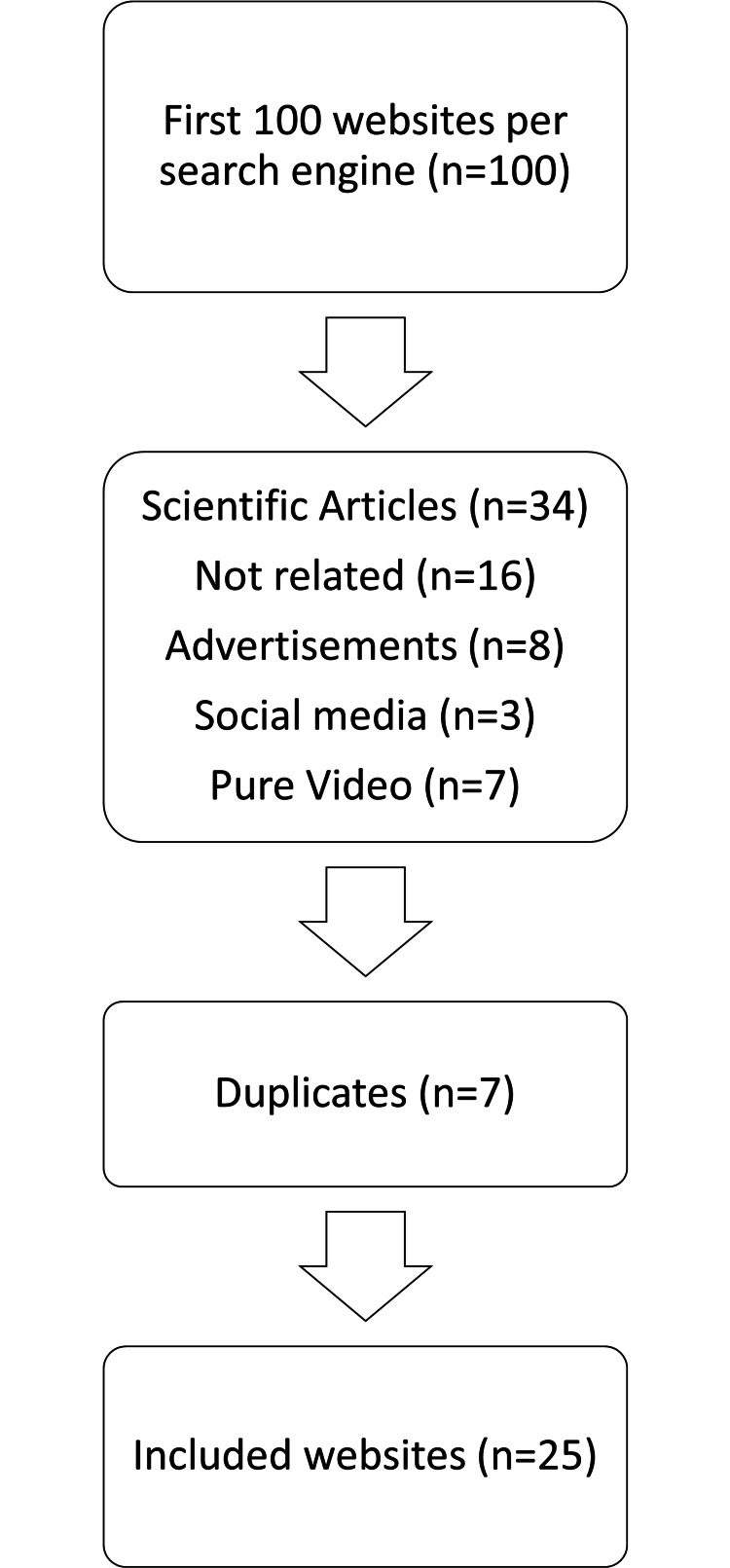
Flow chart of search strategy

Searching on Google.com about the terms treatment of enamel hypomineralization and treatment of hypomineralized teeth revealed 48,500 results; the first 100 websites from the first 10 pages are included. Of these 100 websites, 75 were excluded for the following reasons: 34 were scientific articles, 16 were not related to hypomineralization of teeth, eight contained advertisements and promotional products, three were social media-related, seven were videos, and seven were duplicates. Only 25 websites were selected to be included in the evaluation process; a directory of these sites is available in Appendix 1. The search strategy is summarized in Figure 1.

The websites were categorized based on their affiliation with university/medical centers, which accounted for 10 (40%), with non-profit organizations, which represented five (20%), with commercial entities, which accounted for nine (36%), and with government agencies, which only accounted for one (4%) website. All of the websites were partly related to the treatment of hypomineralization of the teeth. The content type comprised mainly medical facts in 22 (88%) websites, human-interest stories in 18 (72%) websites, and questions and answers in seven (28%) websites. Only one (4%) website featured clinical trials.

The content was presented mainly as a test, with 17 (68%) websites having visual presentations as images, two (8%) websites containing videos, and none featuring an audio presentation. Categorization of websites based on affiliation, specialization, content type, and content presentation is summarized in Table 1.

**Table 1:** Categorization of websites based on affiliation, specialization, content type, and content presentation

Category	Criteria	Number of websites (%)
Affiliation	University/Medical Center	10 (40%)
	Non-profit organization	5 (20%)
	Commercial	9 (36%)
	Governmental	1 (4%)
Specialization	Exclusively related	0 (0%)
	Partly related	25 (100%)
Content type	Medical facts	22 (88%)
	Clinical trials	1 (4%)
	Human interest stories	18 (72%)
	Question and answer	7 (28%)
Content presentation	Image	17 (68%)
	Video	2 (8%)
	Audio	0 (0%)

Quality assessment

The DISCERN scores per question varied, but the mean overall score was 3.08 (± 0.68). The lowest scores related to questions about benefits and risks of treatment (questions 10 and 11) with mean scores of 1.60 (±0.91) and 1.56 (±0.82), respectively. However, the highest score was related to alternative treatments (Question 14), with a mean score of 4.20 (±0.87). The HON code was obtained by only one website. Means and standard deviation scores for DISCERN are summarized in Table 2.

**Table 2:** Means and standard deviation scores for DISCERN

Domain	DISCERN question	Mean (SD)
Reliability	Q1. Explicit aims	3.12 (1.30)
	Q2. Aims achieved	3.44 (1.33)
	Q3. Relevance	3.72 (1.06)
	Q4. Explicit sources	1.80 (1.32)
	Q5. Explicit date	2.84 (1.80)
	Q6. Balanced and unbiased	2.92 (1.35)
	Q7. Additional sources	3.16 (1.11)
	Q8. Areas of uncertainty	2.68 (1.07)
Treatment options	Q9. How treatment works	1.88 (1.39)
	Q10. Benefits of treatment	1.60 (0.91)
	Q11. Risk of treatment	1.56 (0.82)
	Q12. Effects of no treatment	2.20 (1.53)
	Q13. Effects on quality of life	1.88 (1.01)
	Q14. All alternatives described	4.20 (0.87)
	Q15. Shared decision	3.36 (1.19)
Overall rating		3.08 (0.86)

Regarding JAMA benchmarks, authorship and currency were achieved in 10 (40%) websites and 11 (44%) websites, respectively. Based on the number of achieved JAMA items per website, none of the websites had all four items, and only one website had three items. Two items were achieved in seven (28%) websites, and one item was achieved in six (24%) websites. No items were achieved in 11 (44%) of the websites. The quality assessment of the websites is summarized in Table 3.

**Table 3:** Summary of the quality assessment (JAMA & HON Code) of the included websites JAMA: Journal of the American Medical Association, HON: Health on the Net

Item	Frequency (%)
JAMA Benchmarks	
Authorship	10 (40%)
Attribution	3 (12%)
Disclosure	2 (8%)
Currency	11 (44%)
JAMA per website	
No item achieved	11 (44%)
One item achieved	6 (24%)
Two items achieved	7 (28%)
Three items achieved	1 (4%)
Four items achieved	0 (0%)
HON Code	
Obtained	1 (4%)
Not Obtained	24 (96%)

Readability

**Figure 2:**
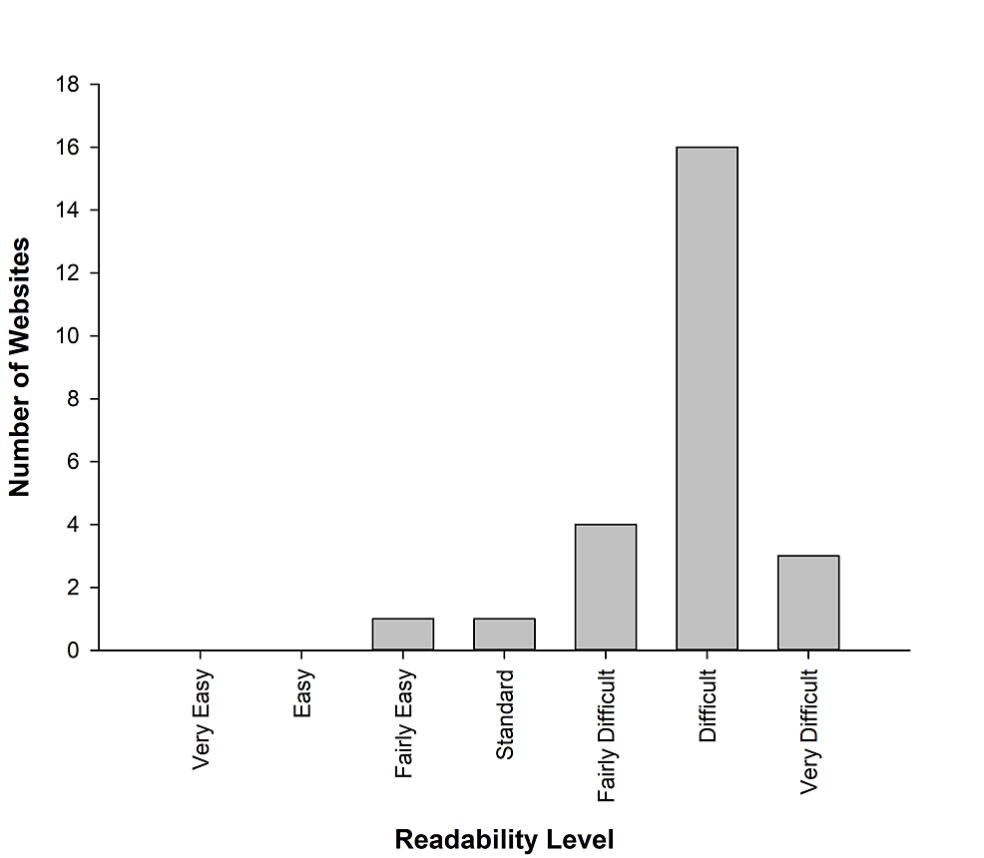
Interpretation of the website readability level according to FRES scale FRES: Flesch Reading Ease Scores

The FRES rating ranged from 9.6 to 74.5 with a mean of 42.1 (± 13.12), while the FKGL had a range, with a minimum score of 7, a maximum score of 16.5, and a mean score of 11.77 (± 2.37). SMOG readability scores ranged from 7 to 14 with a mean of 10.56 (± 1.77). The CLI test yielded a mean score of 12 (± 1.83) with a maximum score of 15 and a minimum score of 7. Based on the FRES, the websites were categorized into difficulties, as shown in Figure 2.

## Discussion

The increase in internet use to access health information about high-occurrence conditions such as enamel hypomineralization highlights the importance of the current study. The number of websites that fulfilled this study’s inclusion criteria was small. Our study’s alignment with Meade and Dreyer's [14] research, where only 21 MIH-related websites were included, underscores the challenges in finding comprehensive resources for specific dental conditions online. The websites included in the current study were categorized as partly related to enamel hypomineralization and not specialized. The overall poor quality and reliability of websites, as indicated by our JAMA criteria assessment, underscores the imperative for the dental health community to enhance online patient educational resources.

Our results showed overall moderate quality of the included website using DISCERN. However, the contents scored poorly for some essential items, including mechanism of action and risk and benefits of enamel hypomineralization treatment options. The results of the JAMA criteria indicated poor quality of the websites, and none of them fulfilled all four criteria. Likewise, several studies investigated websites related to enamel hypomineralization and other dental conditions, such as dental caries and orthodontics, that advocated overall poor quality and reliability [14-16]. This approach emphasizes that the oral health community and dental health care professions should review and improve online patients’ educational resources.

Only one website that has been certified by the HON code met three of four JAMA criteria. To be eligible to display the HON seal on a website, eight criteria must be met, including disclosing author qualifications and maintaining a distinction between advertising and editorial content.

In this research, we employed four criteria to assess the reliability of our results. The FRES scale measures readability, with higher scores indicating easier readability. On the other hand, lower scores of SMOG, FKGL and CLI indicate easier readability. The latter three indicators are commonly used to evaluate the readability of texts and often reflect the educational level in the US or UK school systems. Nevertheless, these indices have also been tested and proven reliable in languages like Hungarian, Portuguese, and Arabic [15,17,18].

The National Institutes of Health (NIH) suggests writing patients' health information in a language that individuals can easily understand in grades six to seven [14]. Our research found that the average FRES, CLI, FKGL and SMOG scores were 42, 12, 11.7, and 10.5, respectively. These scores indicate that the readability level is quite challenging and suitable for students from grade to college level. This complexity could be attributed to terminology and scientific language, mainly since around 40% of the evaluated websites are affiliated with universities or medical centers. Our findings align with studies conducted on medical health-related patient materials [14,16].

Although 17 (68%) of 21 websites assessed in this study presented images as visual aids, only two of the websites presented videos. Using clinical pictures would help the patient identify and recognize different lesions of enamel hypomineralization. However, written content and images may not effectively explain aesthetic treatment options such as bleaching, resin infiltration, and microabrasion. This finding is supported by the notion that treatment option items for the investigated websites displayed low DISCERN scores (< 2). Therefore, using videos could be more effective in explaining the techniques of different treatment modalities [19]. Further research is needed to determine the best way to present treatment methods to public readers.

Poor website quality and difficult readability could misinform patients regarding the diagnosis and treatment options of enamel hypomineralization. This misinformation may negatively affect patient-dentist relationships and create mistrust in professionals’ opinions if it negates what patients read on unreliable websites. This issue could be even more problematic regarding aesthetic conditions such as white and brown discoloration caused by enamel hypomineralization. In contrast, if patients access health information through more reliable sources, they would positively engage in the decision-making process and improve patient-dentist relationships and trust [20]. Hence, the results from this study emphasize the essential role of dental health professionals: first, to be aware of variable online sources and their reliability to be able to discuss with the patient any misinformation. Second, they must actively participate in creating reliable, high-quality online information regarding different types of enamel hypomineralization. Websites’ contents must be easily understandable by the general public at different levels of education. Publishers should consider utilizing videos, visual aids, simple language, and summaries and avoid complex dental terminologies to improve the quality and readability of presented information. The publisher should ensure that the references and authors’ credentials are explicit on the website to give the reader an idea about the presented information’s reliability.

Regarding the limitation of this study, we focused on websites only and did not include information presented on social media. Different social media applications became common sources of information with a profound influence on public health, particularly on aesthetic issues, including enamel hypomineralization [21]. However, the available tools and indices were mainly designed to measure the quality and readability of written documents. In light of our findings, it is imperative that efforts be made to raise awareness among website publishers regarding the importance of HON code certification and to enhance the quality and accuracy of online resources available to patients.

## Conclusions

Evaluating the web-based information related to various dental specialties is essential in recent days. This study assessed the quality and readability of web-based information regarding dental hypomineralization. Most of the websites have a university or medical center affiliation. However, it is partly related to the specialty. Around two-thirds of websites use images to present the content. The online information is inaccurate, poor quality, and challenging for an average person to read. Dental professionals should be aware of this information’s quality and contribute to its improvement. 

## Appendices

**Table 4:** A dictionary of the internet sites used in this study

Websites
https://www.bauersmiles.com/2013/02/enamel-hypomineralization-treatment.html/
https://www.aaronsondds.com/blog/enamel-hypoplasia-hypomineralization-an-explanation-from-your-dentist-in-midtown-east
https://www.comparingexpert.com.au/chalky-teeth-enamel-hypomineralization/
https://www.healthline.com/health/enamel-hypoplasia
https://www.bitesizepediatricdentistry.com/whats-this-spot-on-my-tooth-enamel-hypoplasia-or-hypomineralization/
https://southridgepd.com/weak-tooth-enamel-causes-prevention-and-treatment/
https://www.drugs.com/cg/hypomineralization-of-the-tooth.html
https://happykidsdental.co.uk/preventing-and-protecting/dental-hypomineralisation/
https://www.carefreedental.com/resources/13-dental-health/220-what-is-enamel-hypoplasia
https://doctorstaci.com/enamel-hypoplasia/
https://trustdentalcare.com/what-to-know-about-enamel-hypoplasia-causes-treatment/
https://www.inspiro.org.au/blog/what-to-do-for-your-child-with-hypomineralisation
https://en.wikipedia.org/wiki/Molar_incisor_hypomineralisation
https://www.blog.ivoclar.com/dentist/en/interview-molar-incisor-hypomineralization-a-silent-epidemic-in-children
https://evident.org.au/images/documents/What%20is%20molar%20hypomineralisation%20FINAL.pdf
https://www.kiddiesdentalcare.com.au/23032021info4mih/
http://www.dentistforchildren.com.au/molar-incisor-hypomineralization/
https://www.colgate.com/en-us/oral-health/adult-oral-care/enamel-hypoplasia-hypomineralization-and-teeth-effects
https://www.kids-dentist.com.au/hypomineralised-molars/
https://thetoothcompanykids.co.nz/treatment/specialized-treatments/hypomineralised-teeth-management/
file:///Users/rahafsaud/Downloads/Molar%20Incisor%20Hypomineralisation%20(MIH)_final%20Jun20.pdf+A14
https://sunrisedental.co.uk/wp-content/uploads/2022/01/MIH.pdf
https://greenspointdental.com/hypoplastic-hypomineralized-teeth/
http://lsidesigngroup.com/2016/05/20/2-oral-rinses-to-protect-hypomineralized-teeth-from-bacterial-infections/
https://crest.com/en-us/oral-care-tips/tooth-enamel/enamel-hypoplasia-causes-symptoms-treatment
